# Ultrasound assisted phytochemical extraction of red cabbage by using deep eutectic solvent: Modelling using ANFIS and optimization by genetic algorithms

**DOI:** 10.1016/j.ultsonch.2024.106762

**Published:** 2024-01-09

**Authors:** Kasturi Pusty, Kshirod Kumar Dash, Souvik Giri, G.V.S. Bhagya Raj, Ajita Tiwari, Ayaz Mukarram Shaikh, Kovács Béla

**Affiliations:** a Department of Food Processing Technology, Ghani Khan Choudhury Institute of Engineering and Technology, Malda, West Bengal, India; b Department of Agricultural Engineering, Assam University, Silchar, Assam, India; c Faculty of Agriculture, Food Science and Environmental Management Institute of Food Science, University of Debrecen, Debrecen 4032, Hungary

**Keywords:** Red cabbage, ANFIS, Ultrasonication, Extraction, Genetic algorithm

## Abstract

The present investigation studied the effect of process parameters on the extraction of phytochemicals from red cabbage by the application of ultrasonication and temperature. The solvent selected for the study was deep eutectic solvent (DES) prepared by choline chloride and citric acid. The ultrasound assisted extraction process was modeled using adaptive neuro-fuzzy inference system (ANFIS) algorithm and integrated with the genetic algorithm for optimization purposes. The independent variables that influenced the responses (total phenolic content, antioxidant activity, total anthocyanin activity, and total flavonoid content) were ultrasonication power, temperature, molar ratio of DES, and water content of DES. Each ANFIS model was formed by the training of three Gaussian-type membership functions (MF) for each input, trained by a hybrid algorithm with 500 epochs and linear type MF for output MF. The ANFIS model predicted each response close to the experimental data which is evident by the statistical parameters ($R^{2}>0.953$ and RMSE <1.165). The integrated hybrid ANFIS-GA algorithm predicted the optimized condition for the process parameters of ultrasound assisted extraction of phytochemicals from red cabbage was found to be 252.114 W for ultrasonication power, 52.715 °C of temperature, 2.0677:1 of molar ratio of DES and 25.947 % of water content in DES solvent with maximum extraction content of responses, with fitness value 3.352. The relative deviation between the experimental and ANFIS predicted values for total phenolic content, antioxidant activity, total anthocyanin activity, and total flavonoid content was found to be 1.849 %, 3.495 %, 2.801 %, and 4.661 % respectively.

## Highlights

• Anthocyanins are responsible for the red and purple colors of red cabbage.
• UAE was conducted at ultrasonication power of 100–300 W and temperature 30–60 °C.
• Adaptive Neuro-Fuzzy Inference System integrates fuzzy logic and neural networks.
• Deep eutectic solvents are green and sustainable alternative for extraction of phytochemicals.

## Graphical abstract

**Figure:**
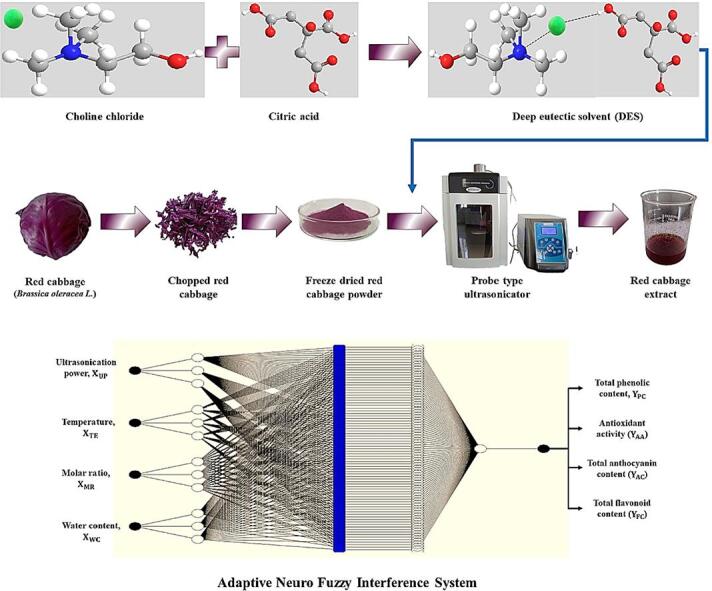

## 1 Introduction

Red cabbage (*Brassica oleracea L.*) is a dark purple colored vegetable (heads) that originated from Europe and belongs to the family Brassicaceae which includes radish (roots), cauliflower (inflorescence), and kale (leaves) [55]. Red cabbage is also identified as purple cabbage and gained popularity all over the world due to easy cultivation and low commercialization cost [58]. The vegetable is a rich source of various bioactive compounds such as dietary fiber, vitamin C and K, minerals, and phenolic compounds like anthocyanins, flavonols, and glucosinolates which are responsible for health benefits [20], [53], [68]. Red cabbage exhibits higher antioxidant activity due to the phytocompounds and was used for therapeutic purposes [50]. Red cabbage has numerous positive health effects especially on the cardiovascular system as it improves capillary blood vessel tightness and prevents the aggregation of thrombocytes in the blood vessel, decreasing the risk of cardiovascular diseases [64]. The red–purple color of red cabbage was due to the presence of water soluble, vacuolar natural pigment anthocyanin which holds more antioxidant activity than the anthocyanin present in different agricultural produce due to its molecular structure and hydroxyl groups are acyl protected provide higher stability to heat and light [28], [78].

Color of the processed foods is one of the main quality attributes which play a vital role in the acceptance of food products by consumers and generally synthetic colorants were used in various food products due to their high stability to different processing conditions [51]. In the present scenario, the consumer’s preference toward healthy foods growing day by day and the utilization of synthetic colorants were found to have a negative impact on health therefore this generated the need for the replacement of synthetic colorants with natural colors extracted from plant sources [24]. The anthocyanin from the red cabbage has the potential to be used as a replacement for one of the harmful red azo dye Allura red (E 129) [63]. Anthocyanin dye is very useful as it gives red, pink, or magenta color in acidic solutions, purple in neutral solutions, and varies from blue to green to yellow in basic solutions [37]. Red cabbage processing generates a vast amount of agro-industry waste which has potential for the extraction of anthocyanin [67].

The phytochemicals from the red cabbage can be obtained by the process of extraction. Choosing the appropriate extraction technique along with process conditions plays an important phase in extracting heat and light sensible chemical compounds from plant sources with the least possible loss to their properties [4]). Different extraction methods that are currently being used in food industry for extraction of various bioactive compounds from agricultural produce were subcritical water extraction method for phenolic compounds from waste onion skin [5]; microwave-assisted extraction method for anthocyanin recovery from blueberry bagasse [25]; ultrasound assisted extraction process for betacyanin from dragon fruit ([7]; polyphenols from olive leaf by supercritical extraction [14].

Among these extraction techniques, ultrasound-assisted extraction (UAE) produces a higher yield of extraction with minimal loss of phytochemical properties [61]). Moreover, the UAE process is eco-friendly and requires less quantity of solvent, easy operation due to less instrumentation, producing higher yields at short extraction time and less temperature [69]. Ultrasound assisted extraction is based on the principle of acoustic cavitation or hydrodynamic cavitation, which is produced by collapsing of microscopic gas bubbles produced during the simultaneous fluctuation of high pressure and low pressure on the solution [41]. This phenomenon increases the mass transfer rate and penetration capability of the solvent which induces the release of intracellular phytocompounds into the solvent [57]. UAE method has been adopted by many researchers in the recent past to extract phytocompounds from different plant based sources such as purple potato [13]; black jamun [70]; mango peel [34]; and grapes [77]. Various factors that influence the extraction yield of phytochemicals during the UAE process include ultrasonication power, ultrasonication frequency, solvent ratio, extraction temperature, extraction time, and type of solvent. Deep eutectic solvent (DES) has emerged as a novel class of green solvents that are widely used in the food industry in recent years due to their eco-friendly non-toxic nature and are effective solvents compared to conventional organic solvents (ethanol, methanol, chloroform, glycerol) for extracting of bioactive compounds from fruits and vegetables [17], [27]. DES is a solvent made of two components at a particular temperature and the two components mainly consist of a quaternary salt as a hydrogen bond acceptor and a hydrogen bond donor [75]. DES have high selectivity for specific compounds, allowing for targeted extraction without co-extracting unwanted substances. Because of their distinct chemical characteristics, DES can increase solubility and hence extraction efficiency.

Adaptive Neuro Fuzzy Interference System is a hybrid data learning technique which was first introduced by Jang, by compiling two machine learning techniques neural network (NN) and fuzzy interference system (FIS) [31], [73]. Neural networks are advanced learning method tools for establishing an interconnection between input–output variables with precision and fuzzy interference system is based on fuzzy IF-THEN rules for predicting complex uncertainties more accurately [54]. Neural networks have the capability of self-learning from the available data whereas fuzzy interference systems are incapable of learning the rules by themselves and require an optimization process of the parameters. Adaptive neuro-fuzzy inference system (ANFIS) integrates the data learning approach of neural networks and heuristic reasoning attribute of fuzzy interference system to apply human like thinking in soft computing. This combination eliminates the disadvantages of both neural networks and fuzzy rules. ANFIS was implemented by many researchers due to its high accuracy in predicting the experimental data [36]. The main objective of the present investigation is to study the effect of ultrasonication power, temperature, molar ratio of dep eutectic solvent, and water content of DES on the total phenolic content, antioxidant activity, total anthocyanin content, and total flavonoid content of the red cabbage extract during UAE extraction of phytochemicals from red cabbage. The UAE process was modeled using an adaptive neuro-fuzzy interference system (ANFIS) and optimized by integrating ANFIS with genetic algorithm (GA).

## 2 Materials and methods

### 2.1 Raw materials

Fresh red cabbage (*Brassica oleracea L.*) was collected from the local market of Malda, West Bengal, India. The whole red cabbage was cleaned and chopped into pieces in a dirt-free environment. The chopped red cabbage was dried in a freeze drier (Lyolab Freeze Lab, Lyophilization Systems Inc., USA) for 24 hr and then ground into powder using a laboratory mixer grinder, sieved through a screen with a mesh size of 250µ and stored in polyethylene bags in a dark place at 4 °C. The chemicals used in the study were analytical grade and procured from Himedia, India. The chemicals used were ethanol (99 % purity), sodium carbonate $\left({Na}_{2}{CO}_{3}\right)$, Folin–Ciocalteu (FC) reagent, gallic acid, DPPH (2,2-diphenyl-1-picrylhydrazyl, Potassium chloride (KCl), Sodium acetate (${CH}_{3}COONa$).

### 2.2 Preparation of deep eutectic solvent (DES)

The DES prepared by choline chloride as the hydrogen acceptor and citric acid as the hydrogen bond donor was reported to be effective for the extraction of anthocyanin rich extract [32]. The DES solvent was prepared according to the procedure described by [72] with little modification [72]. Briefly, all the chemicals were dried in a hot air oven at 45 °C for 1 hr and then mixed according to the molar ratio presented in Table 1.

**Table 1:** Evaluated factors, factor notation, and their levels in CCD design.

**Parameter**	**Ultrasonication power**	**Temperature**	**Molar ratio**	**Water content**
**Unit**	**W**	**°C**	**–**	**%**
**Symbol**	$X_{UP}$	$X_{TE}$	$X_{MR}$	$X_{WC}$
**Maximum**	300	70	2.5	35
**Mean**	200	50	1.5	25
**Minimum**	100	30	0.5	15

### 2.3 Ultrasound assisted extraction of phytochemicals from red cabbage

Ultrasound assisted extraction method of phytochemicals from red cabbage was carried out in an ultrasonic homogenizer with a probe (U500, Takashi, Japan) using a deep eutectic solvent. Freeze-dried red cabbage powder was dissolved in DES solvent in a ratio of 25:1 ml/g in a 60 ml beaker. The extraction was carried out at different conditions of ultrasonication power and temperature for 20 min of extraction time [7].

### 2.4 Experimental design

The independent variables selected for the extraction of phytochemicals from red cabbage during the UAE process were ultrasonication power (100 to 300 W) extraction temperature (30 – 60 °C), molar ratio of the DES solvent (0.5–––2.5), and water content in the DES solvent (15 – 30 %). The power range of 100–––300 W was considered effective for extraction because the power within this range is sufficient to generate and sustain cavitation, facilitating efficient extraction. Within this power range, the ultrasonic waves can be evenly dispersed throughout the sample, ensuring a constant and comprehensive extraction process across the substance being processed. From the preliminary experiments it was also observed that within the 100 to 300 W range, there is a balance between effective extraction and beyond a power level of 300 W there is risk of degradation of bioactive compounds due to excessive energy. Similarly, it was observed that the risk of degrading heat-sensitive compounds in the sample was minimized within the temperature range of 30–60 °C. Each independent variable was varied for 5 levels and designed using central composite design CCD) to study their effect on the total phenolic content ($Y_{PC}$), antioxidant activity ($Y_{AA}$), total anthocyanin content ($Y_{AC}$), and total flavonoid content ($Y_{FC}$) of the UAE red cabbage extract by ANFIS modelling (Table 1). The CCD design yielded a total of 30 experiments with different combinations of independent variables.

### 2.5 Total phenolic content

The total Phenolic content of red cabbage extract was estimated using Folin–Ciocalteu (FC) reagent and spectrophotometric method [1]. Briefly, UAE red cabbage extract of 0.5 ml was mixed with 2.5 ml 10 % FC reagent and incubated for 5–7 min. Then 7.5 %(w/v) sodium carbonate (${Na}_{2}{CO}_{3}$) solution of 2.5 ml was added to the mixture, the solution volume was made up to 10 ml with distilled water and incubated for 30 min at room temperature. After incubation absorbance was measured at 765 nm against the blank in a spectrophotometer. The calibration curve was prepared by different concentrations of gallic acid and a similar procedure was followed for the determination of absorbance. Total phenolic content ($Y_{PC}$) was expressed in terms of gallic acid equivalents (mg GAE/g d.w.) and calculated using the equation presented in Eq. (1). Each experiment is performed three times and average data was taken to represent total phenolic content.

(1) $$Total\;phenolic\;content\left(Y_{\mathrm{PC}}\right)=\frac{c\times V}{w}$$

where, c = concentration of gallic acid obtained from calibration curve in mg/mL, V = volume of red cabbage extract in mL, and w = mass of extract in gram.

### 2.6 Antioxidant activity

The antioxidant activity of the UAE red cabbage extract was quantified using DPPH (2,2-diphenyl-1-picrylhydrazyl) free radical scavenging activity method [62]. This method is based on the principle of the ability of the antioxidant to scavenge free radicals from the solution which causes discoloration of the DPPH solution from purple to pale yellow. UAE red cabbage extract of 0.5 ml was mixed with 3 ml of DPPH solution and incubated for 30 min in a dark place. After 30 min, absorbance was measured at 517 nm against a blank using UV-spectrophotometer. Antioxidant activity was expressed in terms of the inhibition percentage of DPPH scavenging and calculated according to the equation Eq. (2).

(2) $$Antioxidant\;activity\;\left(Y_{AA}\right)=\left(\frac{A_{\mathrm{blank}}-A_{\mathrm{sample}}}{A_{\mathrm{blank}}}\right)\times 100$$

where, $A_{\mathrm{blank}}$ = absorbance of the blank sample, $A_{\mathrm{sample}}$ = absorbance of the sample with red cabbage extract.

### 2.7 Total anthocyanin content

Anthocyanin Content was measured using spectrophotometric pH differential method [74]. Red cabbage extract was adjusted to a buffer of pH 1 and pH 4.5 with potassium chloride (0.025 $\mathrm{mol}L^{-1}$) and sodium acetate (0.5 $\mathrm{mol}L^{-1}$) respectively. Buffer solution was incubated for 15 min in a dark place at room temperature. The absorbance was recorded at 510 nm and 700 nm. Total Anthocyanin content ($Y_{AC}$) was calculated using the equation Eq. (3) and Eq. (4), expressed as $mg\;g^{-1}$ dry matter.

(3) $$A={\left(A_{510}-A_{700}\right)}_{pH1}-{\left(A_{500}-A_{700}\right)}_{pH4.5}$$

(4) $${Anthocyanin\;content\;(Y}_{AC})=\frac{A\times m_{w}\times DF}{\varepsilon \times l}\times 1000$$

where, A = absorbance obtained from equation, $m_{w}=$ molecular weight of anthocyanin (449.2 g mol^−1^), DF = dilution factor, ε= extraction coefficient (26,900 L cm^-1^mol^−1^), l= path length (1 cm).

### 2.8 Total flavonoid content

The total phenolic content of UAE red cabbage extract was quantified using spectrophotometry method according to the procedure described with little modifications [85]. Briefly, UAE red cabbage extract of 0.5 ml was mixed with 0.1 ml of 5 % ${NaNO}_{2}$ and incubated for 5 min, thereafter 0.1 ml of 10 % $AlCl_{3}$ was added to the mixture. The mixture was mixed well for 1 min and then 1 ml of NaOH was added quickly. The mixture absorbance was measured at 510 nm using UV-Spectrophotometer. For the calibration cure different concentrations of quercetin (QE) standard solution was prepared and a similar procedure was followed for mixing and measuring the absorbance. The total flavonoid content of red cabbage extract was expressed in mg of QE/g red cabbage dry weight.

### 2.9 ANFIS modeling

**Fig. 1:**
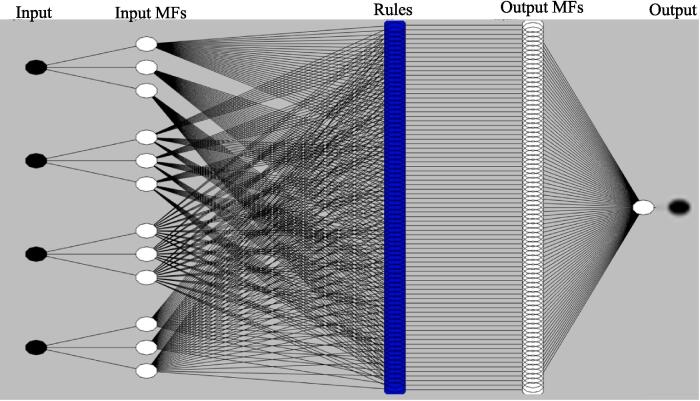
ANFIS architecture proposed for the ultrasound assisted extraction process.

The ultrasound assisted extraction of phytochemicals from red cabbage was modeled using ANFIS according to the procedure described by Ogaga Ighose et al., [56]. The ANFIS model was designed by MATLAB (MATLAB 2018) with Fuzzy Logic Toolbox and the proposed ANFIS architecture was presented in Fig. 1, where it consists of five layers. The obtained 30 experimental sets of data consisting of input and output variables were divided into two parts containing 70 % and 30 % used for the training and checking of the ANFIS model respectively. Each data set consisted of four independent variables as inputs and one dependent variable was used in output at a time. Three membership function nodes were assigned for each of the independent variables. The experimental data were fitted into a first order Takagi-Sugeno model to train the ANFIS model. The input and output membership functions (MFs) were selected based on the lower RMSE value during training of the ANFIS and two methods were implemented namely backpropagation and hybrid (a forward pass and a backward pass) approach for training.

The first layer is known as the fuzzy layer where each node is an adaptive node with node function or membership functions (MFs) that allow fuzzification of the input parameter, second layer is the product layer where each node (non-adaptive) displays the firing strength of a rule by multiplication, third layer is the normalized or rule layer here every node signifies the normalized firing strength of each rule, the de-fuzzy layer is the fourth layer each node at this layer are adaptive and represents the product of the first-order Sugeno-type polynomial and the normalized firing strength, and fifth layer is the overall summation layers that consist of a single node which is the sum of all the incoming signals [40]. The relative influence of process parameters on the response was calculated according to the procedure described by Dash et al., [19].

### 2.10 Multi-objective genetic algorithm

The output of the ANFIS model was integrated with genetic algorithm (GA) for the optimization of the model. GA is based on the ‘survival of the fittest’ strategy by means of selection, reproduction, crossover, and mutation of the initial population ([6]. The fitness function (FF) used for the optimization of the UAE process was presented in Eq. (5)

(5) $$FF=\left\{\begin{array}{c} \max Y_{PC}\left(X_{UP},X_{TE},X_{MR},X_{WC}\right)\\ \max Y_{AA}\left(X_{UP},X_{TE},X_{MR},X_{WC}\right)\\ \max Y_{AC}\left(X_{UP},X_{TE},X_{MR},X_{WC}\right)\\ \max Y_{FC}\left(X_{UP},X_{TE},X_{MR},X_{WC}\right)\\ 100W\leq X_{UP}\leq 300W\\ 30^{{}^{\circ}}C\leq X_{TE}\leq 70^{{}^{\circ}}C\\ 0.5:1\leq X_{MR}\leq 2.5:1\\ 15\%\leq X_{WC}\leq 35\% \end{array}\right)$$

### 2.11 Statistical analysis

The statistical analysis used in the present study for validating the adequacy of the ANFIS model was the coefficient of determination ($R^{2}$) using Eq. (6), root mean square error (RMSE) using Eq. (7), and relative deviation percentage using Eq. (8) [8].

(6) $$R^{2}=1-\frac{\sum_{i=1}^{n}\left(Y_{p}-Y_{e}\right)}{\sum_{i=1}^{n}\left(Y_{a}-Y_{e}\right)}$$

(7) $$RMSE=\sqrt{\frac{\sum_{i=1}^{n}\left(Y_{p}-Y_{e}\right)}{n-1}}$$

(8) $$R_{d}=\frac{100}{n-1}\sum_{i=1}^{n}\frac{\left|{Y_{e}-Y}_{p}\right|}{Y_{e}}\;$$

where $Y_{p}$ is the predicted value from the model, $Y_{e}$ is the observed value or experimental value, $Y_{a}$ is the average response value, and n is the number of experiments conducted.

## 3 Results and discussion

### 3.1 ANFIS modeling of UAE of red cabbage

The ultrasound assisted extraction of phytochemicals from red cabbage using deep eutectic solvent was successfully modeled by the ANFIS method and the best architecture of ANFIS with four independent variables ultrasonication power (W), temperature (°C), molar ratio, water content (%) for predicting the responses total phenolic content (mg GAE/g d.w.), total anthocyanin content (mg/g d.w.), antioxidant activity (% DPPH inhibition) and total flavonoid content (mg QE/g d.w.) was illustrated in Fig. 1. ANFIS formed by the training of three Gaussian type membership function for each input, trained by hybrid algorithm with 500 epochs and linear type MF for output MF found to have lower root mean square error (RMSE) and higher $R^{2}$. The formed ANFIS consists of 81 rules resulted high accurate predictions for all of the four responses. The RMSE values for the ANFIS model formed for response total phenolic content ($Y_{PC}$), antioxidant activity ($Y_{AA}$), total anthocyanin content ($Y_{AC}$), and total flavonoid content ($Y_{FC}$) was found to be 0.131, 1.165, 0.009, and 0.003 respectively. For the same sequence of response, the $R^{2}$ value was found to be 0.954, 0.953, 0.964, and 0.992 respectively. Similar results of higher $R^{2}$ value and a lower MAE value was reported for predicting moisture ratio by ANFIS formed by Gaussian as input MF, linear as output MF, hybrid type of learning algorithm, 1000 epoch, and three MF for the three independent variables in input during hot air-rotary drum drying of green pea for predicting moisture ratio [35].

### 3.2 Effect of independent variables on total phenolic content of red cabbage extract

The total phenolic content of red cabbage extract was in between 4.154–––8.163 mg GAE/g d.w. The relative effect of process parameters on the response total phenolic content predicted by the ANFIS model was presented in Table 2. According to Table 2, the three process parameters ultrasonication power, molar ratio, and water content were found to have a positive influence while the temperature had a negative effect. The positive effect signifies the increase of process parameters increased the response while the negative effect implies a decrease in response with an increase of process parameters. Among these process variables molar ratio (0.740) showed a higher effect followed by ultrasonication power (0.630), temperature (0.380), and water content (0.351). The actual $Y_{PC}$ values at different combinations of process parameters and predicted values by the formed ANFIS for $Y_{PC}$ at respective combinations of process parameters presented in Fig. 1(i) and found to have a close agreement between them indicated by a correlation value close to unity.

**Table 2:** Relative effect of process parameters on responses of UAE red cabbage extract.

**Process variable**	$y_{PC}$	$y_{AC}$	$y_{AA}$	$y_{FC}$
$x_{UP}$	0.630	1.021	0.701	1.144
$x_{TE}$	−0.380	−0.399	−0.867	0.476
$x_{MR}$	0.740	0.925	0.649	0.255
$x_{WC}$	0.351	−0.678	0.204	−1.647

**Fig. 2:**
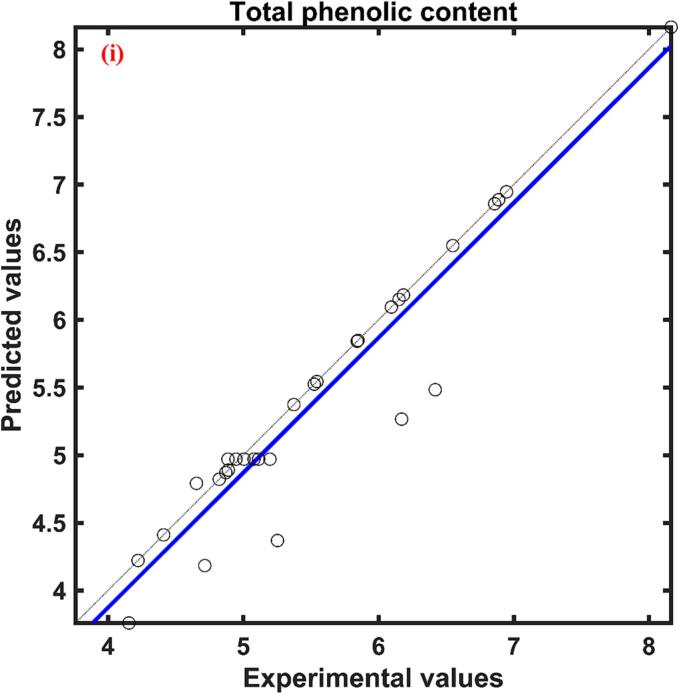

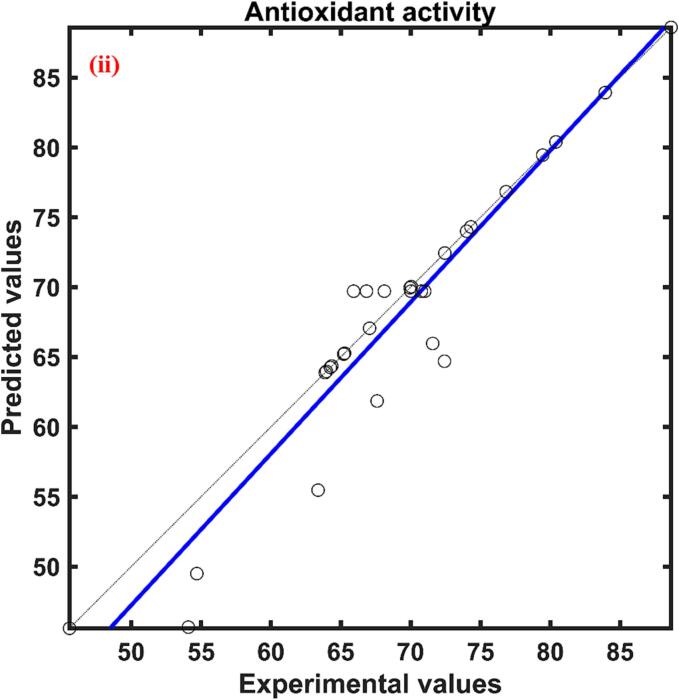

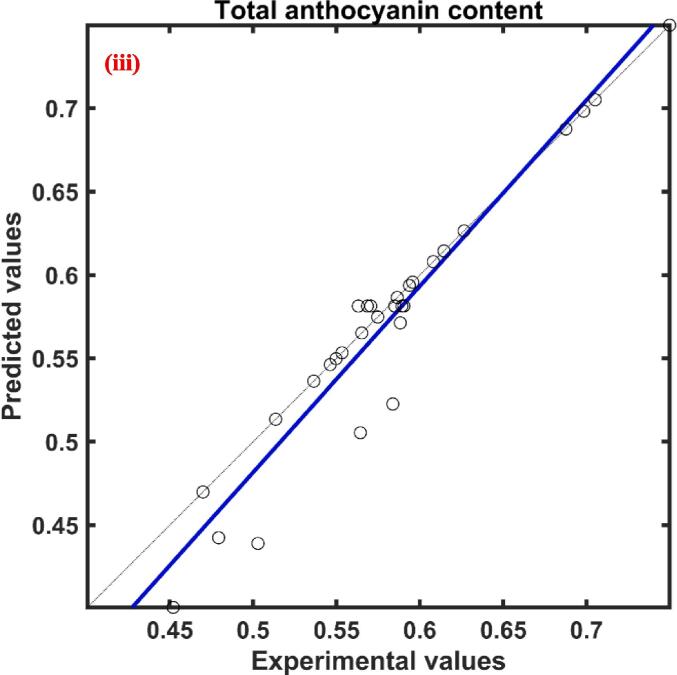

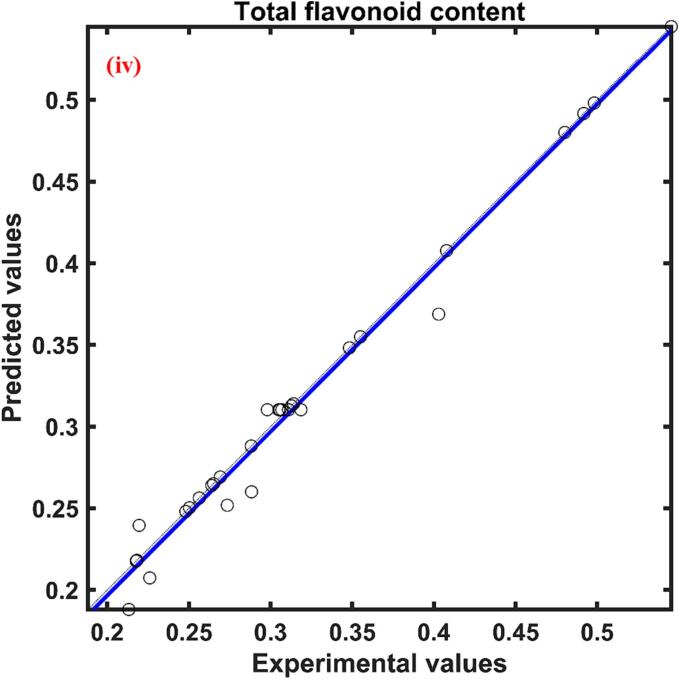
Experimental Vs ANFIS model predicted values for (i) Total phenolic content; (ii) Antioxidant activity; (iii) Total anthocyanin content; and (iv) Total flavonoid content of UAE red cabbage extract. (For interpretation of the references to color in this figure legend, the reader is referred to the web version of this article.)

**Fig. 3:**
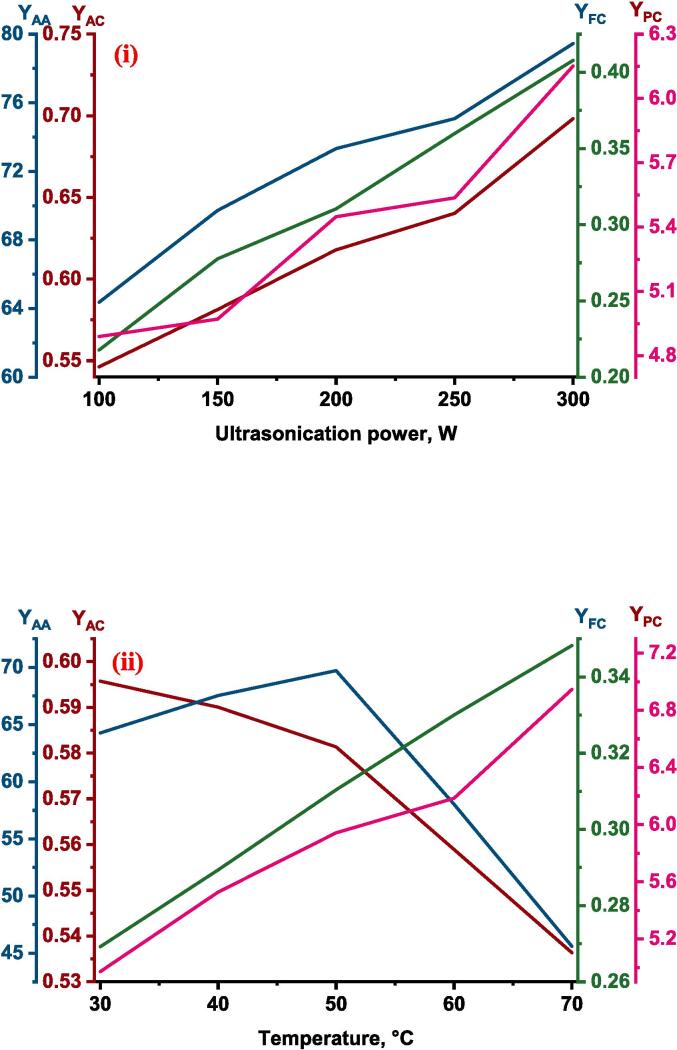

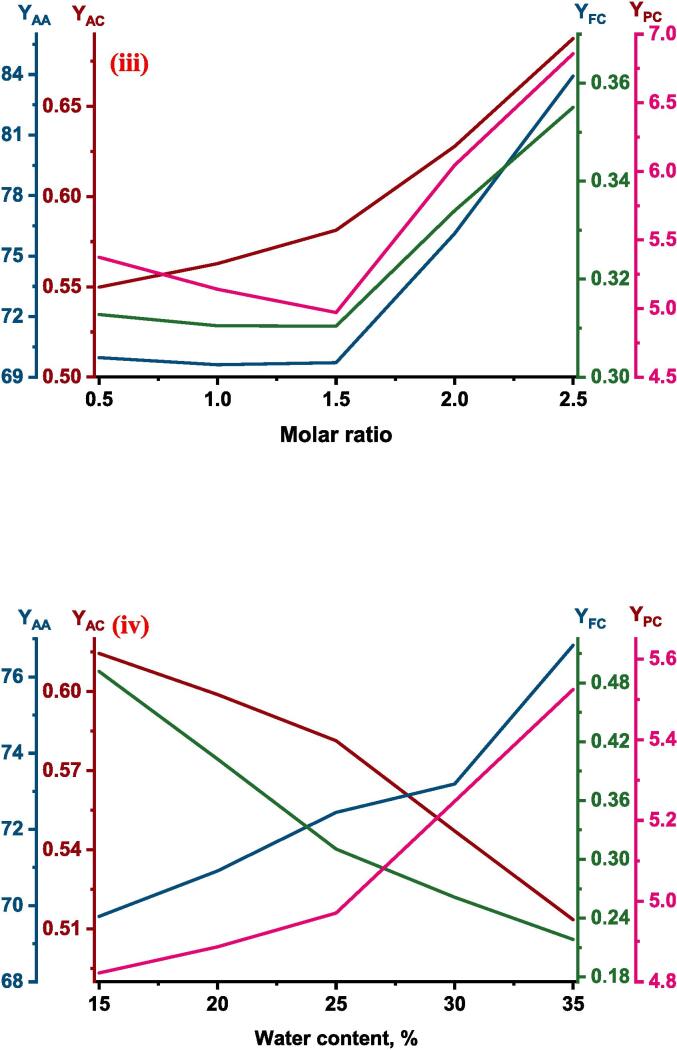
Single factor effect of process variables (i) Ultrasonication power; (ii) Temperature; (iii) Molar ratio of DES; and (iv) Water content in DES on the responses of UAE red cabbage extract. (For interpretation of the references to color in this figure legend, the reader is referred to the web version of this article.)

The rise of ultrasonication power caused an increase in the $Y_{PC}$ of the red cabbage extract, which is evident in Fig. 2(i). Higher power levels contribute to increased movement and agitation within the liquid, which enhances mass transfer. This movement facilitates the dispersion of solutes from the solid matrix into the solvent, hence enhancing the efficiency of extraction ([87]. The higher ultrasonication power might be adequate to overcome the static pressure at the tip of the ultrasonication probe and this further elevates the turbulent tremor capable of rising the ultrasound effect for higher yield during extraction [60]. Comparable results of an increase in total phenolic content from 4.48 ± 0.12 mg GAE/ml to 8.52 ± 0.19 mg GAE/mL with the rise in ultrasonication power from 200 to 600 W during ultrasound assisted extraction from blueberry juice operated at ultrasonication frequency of 20 kHz, 60 °C temperature for 15 min of extraction time [80]. Similar result of an increase in phenolic acids like caffeic from 36.9 to 57.0 µg/g of d.w., sinapic from 124.0 to 155.6 µg/g of d.w., p-cinnamic from 79.6 to 106.8 µg/g of d.w., vanillic from, 28.8 to 32.6 µg/g of d.w., with an increase of ultrasonication power from 3.2 to 30 W was reported during ultrasound assisted extraction of phenolic compounds from citrus peel operated at a frequency of 60 kHz and temperature of 15 °C for 20 min of extraction time ([45]. The negative influence of temperature on phenolic content may be due to the heat lability of the polyphenols where these compounds get denatured at higher temperatures[23], [39]. Another reason for the decrease in $Y_{PC}$ at higher temperatures might be due to the fact of decrease in vapor pressure of the solution which results in a lower sonication effect [15]. So, the results, suggest that the phenolics content present in the red cabbage was unstable at higher temperatures and the decrement of total phenolic content can be visualized by the single factor analysis presented in Fig. 3(ii) This is in agreement with [3], where a decrease in $Y_{PC}$ from 30.33 ± 0.27 to 18.74 ± 0.13 mg GAE/g d.m. was reported in response to rise in temperature from 20 to 60 °C during ultrasound-assisted extraction of phenolics from blueberry pomace [3]. Similarly, [86] also observed of 6 % decrease in extraction yield with an increase in temperature from 30 to 50 °C during ultrasound-assisted extraction of oil from flaxseed [86].

A positive relative influence on total phenolic content of the red cabbage extract was displayed by the molar ratio of DES demonstrated by the single factor analysis presented in Fig. 3(iii). This increment in $Y_{PC}$ can be attributed to the fact that phenolics of plant materials were found to be highly connected with H bonding, ionic charge, and polarity that are intrinsic to DES nanostructure, which is influenced by the molar ratio [21]. A strong hydrogen bond network formed between DES components and solute as the availability of H-bond donors increased with the increment of molar ratio which induces the dissolution of phenolic compounds in DES which results in the improvement of extraction efficiency of phenolic compounds from the extract [83], [84]. Identical trends of increase of phenolic compounds from 70 to 100 % with an increase of molar ratio from 1:1 to 2:1 of DES formed by chloride-oxalic acid and oxalic acid were reported during ultrasonic assisted extraction of polyphenols from *Aegle marmelos choline* [66]. The negative influence of water content in DES (Fig. 3(iv)) may be possibly due to the highly hydrophobic nature of red cabbage phenolic compounds resulted in the lower solubility at higher water content. In order to dissolve and extract compounds, DES usually depends on hydrogen bonding interactions between its components. Higher water content can disrupt these interactions, reducing the ability of the DES to solubilize target compounds, especially those that might be more soluble in the less polar environment of the pure DES. Another viable reason might be associated with the viscosity of DES solvent where water content is incapable to reduce the viscosity of the solvent that in turn caused a decrease in mass transfer [12]. Comparable trends of decrease in total phenolic content (mg GAE/g d.w.) from 61.21 to 50.41 was reported with an increase of water content (%) in DES from 10 to 50 during extraction of phenolics from rosemary leaves at a temperature of 25 °C and liquid to solid ratio of 40:1 ml/g by using DES formed by choline chloride and 1,2-propanediol in the molar ratio 1:2 [79].

### 3.3 Effect of independent variables on antioxidant activity of red cabbage extract

The maximum and minimum antioxidant activity (%DPPH inhibition) of the red cabbage extract under different combinations of process parameters was found to be 88.639 and 45.588, illustrated in Fig. 2(ii) As per the ANFIS established relative effect (Table 2), the least effected process parameter was found to be the water content of the DES with the value 0.204 and found to have positive impact on the antioxidant activity of the red cabbage extract. The process parameter ultrasonication power (0.701) and the molar ratio of DES (0.649) were also found to have a positive influence while the temperature (-0.867) was found to have a negative effect on the antioxidant activity of the red cabbage extract, supported by the Table 2. The most prominent process factor affecting the antioxidant activity was temperature followed by ultrasonication power and molar ratio, which can be visualized by the magnitude of the relative effect values. The proposed ANFIS model predicted the antioxidant activity of experiment data with higher accuracy, which was supported by the statistical parameter correlation coefficient and the plot between the actual and predicted values was illustrated in Fig. 2(ii). Influence of ultrasonication power on antioxidant activity has a positive impact as predicted by the ANFIS model and supported by the single factor analysis shown in Fig. 3(i). Polyphenolic compounds are known for their high antioxidant potential as the resonance effect of localized phenolic rings causes various oxidation reduction reactions [22]. As previously mentioned, ultrasonication power increased the extraction rate of polyphenolic compounds from the red cabbage into the DES solvent which might result in higher antioxidant properties of the extract. Similar results of direct relation to phenolic compounds in the extract and antioxidant activity of the extract were reported during ultrasound assisted extraction of phytochemicals from the skin of the grapes [47]. Predicted values of the ANFIS model showed that the influence of temperature on the antioxidant activity of the red cabbage extract has an inverse effect and found to be the have highest influence among other independent process variables. Antioxidant activity decreased with a rise in temperature because of the thermolabile properties of the polyphenolic compounds present in the red cabbage extract. The results were in agreement with Dadi et al., [18] where the antioxidant activity reduced from 336.5 ± 1.8 to 220.7 ± 1.4 mg/g in response to the rise in temperature from 40 to 50 °C during ultrasound assisted extraction of bioactive compounds of *Moringa stenopetala* leaves [18]. The increase in the molar ratio of DES solvent improved the antioxidant activity of the UAE red cabbage extract evidenced by Fig. 3(iii). The improved antioxidant activity with the rise of the molar ratio of DES solvent may be due to the reduction of the viscosity and surface tension of the mixture, resulting in enhancing the mass transfer rate and dissolution of bioactive compounds [82]. Modifying the molar ratio of DES solvent also leads to changes in the hydrogen bonding network and interactions within the solvent, which in turn affects its ability to interact with antioxidant molecules. This alteration facilitates improved extraction or stabilization of antioxidant compounds. Comparable results of enhanced antioxidant activity were reported with an increase of molar ratio of DES solvent prepared using sodium acetate and lactic acid during ultrasound assisted extraction of bioactive compounds from alkanet [83], [84]. Results of relative effect according to the ANFIS model, revealed that the water content of DES influenced antioxidant activity positively and moreover similar trend was followed in Fig. 3(iv). This may be due to the fact that increasing the polarity of the solvent suitable for the solubility of the compounds responsible for antioxidant activity [59]. An increase in antioxidant activity with an increase in water content can also be associated with the reduction of solvent viscosity which might result in less interaction between the solvent and the antioxidant exhibiting compounds (N. [88], [42] observed the antioxidant activity of Mangifera pajang fruit was 11 mg AEAC/g during the solvent extraction method by DES solvent prepared by choline chloride and glycerol in the molar 1:2 with 10 % water content and increase of water content to 20 % at similar conditions of extraction resulted in approximately 4.2 % increase in antioxidant activity [42].

### 3.4 Effect of independent variables on total anthocyanin content of red cabbage extract

The maximum total anthocyanin content under different experimental conditions during ultrasound assisted extraction of red cabbage was found to be 0.750 mg/g d.w. while the minimum total anthocyanin content was 39.71 % lower than the maximum total anthocyanin content of red cabbage extract. According to the relative influence of the independent variable predicted by the formed ANFIS model on the total anthocyanin content of red cabbage extract during the UAE process, ultrasonication power and molar ratio were found to have a positive impact while extraction temperature and water content had a negative effect. The experimental $Y_{AC}$ along with ANFIS predicted values of $Y_{AC}$ were plotted against each other presented in Fig. 2(iii) From the correlation coefficient, which is close to unity it is evident that there is a close agreement between observed and predicted values of $Y_{AC}$. Out of four independent variables, ultrasonication power (1.021) had the most impact on the extraction of anthocyanins from red cabbage while temperature (-0.399) had a lesser impact which is noticeable by the magnitude, presented in Table 2.

The higher anthocyanin content in red cabbage extract obtained at higher ultrasonication power can be attributed to the enhanced movement of the solvent into the intercellular matrix due to the destruction of the cell membrane. High ultrasonication power boosts the effect of thermal, cavitation, and mechanical that causes shorten time for the collapse of bubbles and improves the intensity of cavitation resulting in the destruction of cell membrane which enhance the mass transfer rate for rapid extraction of anthocyanins[4], [52]. These phenomena of ultrasonication power on the solution improve the movement of the molecules and enhance the penetration capability of the solvent into the solute matrix that results in the higher dissolubility of bioactive compounds into the solvent [81]. Identical results of increased total anthocyanin content from 18 to 24.1 C3GE/g d.w. with the rise of ultrasonication power from 60 to 300 W were reported during the ultrasound extraction of anthocyanin from blueberry pomace [26]. Temperature showed a negative effect on $Y_{AC}$ of the red cabbage extract as anthocyanins are heat-labile compounds. Higher temperature activates the coupling effect of oxygen molecules to the solvent which causes the degradation of anthocyanin [29]. Moreover, the rise in temperature also has a negative influence on the cavitation intensity of the ultrasonication process [65]. Hu et al., [30] observed a significant reduction of anthocyanin content from 10.12 mg/g to less than 9 mg/g with an increase of temperature from 50 to 70 °C during ultrasound assisted extraction of anthocyanin from blueberry pomace with ultrasonication frequency of 80 kHz [30]. A similar effect of temperature on the yield of total anthocyanin content was also reported by Liu et al., [43] where the rise in temperature from 25 to 80 °C reduced the extraction yield of total anthocyanin content during the extraction of anthocyanin from blueberry at different combinations of pH and temperature [43].

The molar ratio influences the diffusion rate, mass transfer, polarity and pH and other properties of the DES which in turn affect the extraction yield of anthocyanin from fruits and vegetables [10]. After the ultrasonication power, the molar ratio (0.925) was found to have a higher impact on the total anthocyanin content of red cabbage extract. In the present study, the total anthocyanin content of the red cabbage extract was improved by increasing the molar ratio of the DES. Comparable trends of increase in total anthocyanin content from 39.11 to 42.55 mg/g were reported with an increase of the molar ratio of DES prepared with citric acid and maltose increased from 2:1 to 4:1 during DES extraction of anthocyanin from grape skin [32]. The water content of DES was found to have a negative effect on the extraction yield of anthocyanins from red cabbage during the UAE process which was supported by the relative influence predicted by the proposed ANFIS model with a value of −0.678 and this parameter was found to have lower influence compared with ultrasonication power and molar ratio of DES. Excessive water content can sometimes lead to phase separation in the DES system, forming separate aqueous and DES phases. This phase separation can hinder the extraction process, making it less efficient and reducing the overall yield of anthocyanin. A comparable influence of molar ratio and water content of DES was reported during phytochemical extraction from roselle using DES solvent formed by the combination of sodium acetate and formic acid [84], [83]. The negative effect of the water content of DES might be due to the fact that adding too much water to the eutectic solvent excessively increases the polarity of the solvent and sever hydrogen bonds between the solvent and the anthocyanin compounds [38]. Anthocyanin content decreased after a 25 % increase of water content was observed by Bosiljkov et al., [11] during the extraction of anthocyanin from wine lees by DES and ultrasound assisted extraction [11].

### 3.5 Effect of independent variables on total flavonoid content of red cabbage extract

Total flavonoid content extracted from the red cabbage during ultrasound assisted extraction process on varying different independent variables ranged from 0.218 to 0.492 mg QE/g d.w. Fig. 2(iv). The relative influence calculated by the proposed ANFIS model for total flavonoid extraction was presented in Table 2. According to Table 2, the water content in DES was the most influent parameter when compared with the other process parameters and influenced total flavonoid content in a negative way evidenced by the value −1.647. Whereas the rest of the three process parameters showed positive influence in the descending order as follows ultrasonication power (1.144), temperature (0.476), and molar ratio (0.255). The experimental data was plotted against the ANFIS predicted total flavonoid content data and found to have a close agreement with each other, illustrated in Fig. 2(iv).

The effect of ultrasonication power on the total flavonoid content of the red cabbage was positive as high ultrasonication power resulted in higher flavonoid content due to higher extraction of flavonoids from the matrix due to the positive effect of power. Total flavonoid content was increased with an increase of ultrasonication power as ultrasonic waves of high amplitude are generated by higher ultrasonication power which induces the cavitation process in the ultrasound assisted extraction, resulting in an improvement of extraction efficiency of flavonoids present in the plant materials [46]. This effect has also been confirmed by Wang et al., [76] during ultrasound assisted extraction of flavonoids from olive (Olea europaea) leaves as flavonoid content has increased from 71.52 to 79.03 mg/g in response to the increasing ultrasonication power from 180 to 270 W [76]. The positive effect of temperature on the total flavonoid content of the UAE extract may be due to the heat stability of flavonoids present in the red cabbage extract at higher temperatures. Moreover, molecular movement and solubility of the solvent also increased due to the rise in temperature which influenced the dissolution of flavonoids into solvent [33]. A similar tendency of temperature on flavonoid content was also reported by Ciric et al., [16] during ultrasound-assisted extraction of flavonoids from garlic [16] and during ultrasound assisted extraction of flavonoids from cocoa shell [48].

The molar ratio of DES interacted positively with the total flavonoid content of the red cabbage extract due to the change in density and viscosity of the solvent. Increasing the molar ratio resulted in an increase in extraction efficiency as the changes in viscosity within the solvent increased the mass transfer rate [44]. Identical results of an increase in the molar ratio of DES formed by acetylcholine chloride and lactic acid from 1:1 to 2:1 improved the extraction yield of quercetin (flavonoid) from 75.9 % to 90.1 % in the onion sample [2]. Water content in DES showed a negative influence on the total flavonoid content of the red cabbage extract which can be attributed to the change in the polarity of the solvent which is not a suitable environment for the less polar flavonoid in the extraction. Excessive water content might have decreased the interaction between DES and flavonoid compounds resulted in lower flavonoid efficiency [9]. Hydrogen bonds present in DES also get denatured by higher water concentration in the solvent which results in the degradation of the DES structure and the solubility of the flavonoid compounds is reduced[49].

### 3.6 Optimization by genetic algorithm

The output of the ANFIS model was fed to genetic algorithm and integrated algorithm yielded 18 sets of solutions according to the fitness function given in Eq. (4) The optimum condition was selected based on the fitness value. The optimized condition for the process parameters of ultrasound assisted extraction of phytochemicals from red cabbage was found to be 252.114 W for ultrasonication power, 52.715 °C of temperature, 2.0677:1 of molar ratio of DES, and 25.947 % of water content in DES solvent, with fitness value 3.352. The predicted values of responses' total phenolic content ($Y_{PC}$), antioxidant activity ($Y_{AA}$), total anthocyanin content ($Y_{AC}$), and total flavonoid content ($Y_{FC}$) of the UAE red cabbage at the optimum condition was found to be 7.388 mg GAE/g d.w., 92.286 % DPPH inhibition, 0.757 mg/g d.w., 0.466 mg QE/g d.w. respectively. For the validation of the hybrid ANFIS-GA algorithm, experiments were conducted in triplets at the optimum condition and the responses as the above-mentioned sequence was observed to be 7.527 mg GAE/g d.w., 89.169 %DPPH inhibition, 0.736 mg/g d.w. and 0.489 mg QE/g d.w. respectively, presented in Table 3. The relative deviation between the experimental and predicted values at the optimum condition was found to be less than 5 % which signifies that there was a good agreement between the experimental and predicted values. Comparable findings were obtained in the genetic algorithm optimization of UAE of phenolic compounds from cashew apple bagasse as well as in the UAE of pomegranate peel [89], [90].

**Table 3:** Integrated ANFIS-GA model predicted responses and experimental values at the optimum condition.

**Response**	$Y_{PC}$**,** **mg GAE/g d.w.**	$Y_{AC}$**,** **mg/g d.w.**	$Y_{AA}$**,** **%DPPH inhibition**	$Y_{FC}$**,** **mg QE/g d.w.**
**Predicted**	7.388	0.757	92.286	0.466
**Experimental**	7.527	0.736	89.169	0.489
**Rd, %**	1.849	2.801	3.495	4.661

## 4 Conclusion

The phytocompounds from the red cabbage was successfully extracted by the application of ultrasonication. The total phenolic content, antioxidant activity, total anthocyanin content, and total flavonoid content were found to be in the range of 4.154–––8.163 mg GAE/g d.w., 45.588 – 88.639 %DPPH inhibition, 0.452 – 0.750 mg/g d.w., and 0.213 – 0.545 mg QE/g d.w. respectively under different combinations of process parameters ultrasonication power (100 to 300 W) extraction temperature (30 – 60 °C), molar ratio of the DES solvent (0.5–––2.5), and water content in the DES solvent (15 – 30 %). The extraction process was modeled by using ANFIS and optimized by integrating ANFIS and genetic algorithm. The ANFIS algorithm was found to successfully model the extraction process with higher accuracy and lower error evident by the $R^{2}\left(>0.953\right)$ and RMSE (<1.165) values. The integrated ANFIS-GA model predicted the optimum values of process parameters at 252.114 W for ultrasonication power, 52.715 °C of temperature, 2.0677:1 of molar ration of DES, and 25.947 % of water content in DES solvent, with fitness value 3.352. The practical importance of UAE is based in its ability to maximize extraction processes, improve yield and quality and provide a more sustainable and effective technique of extracting bioactive compounds. Therefore, the research would pave the way for improving ultrasound-assisted bioactive component extraction from a variety of natural resources, increasing the potential uses of these compounds in the food, cosmetics, and pharmaceutical industries.

## Funding

Project No. TKP2021-NKTA-32 has been implemented with support from the National Research, Development, and Innovation Fund of Hungary, financed under the TKP2021-NKTA funding scheme.

## CRediT authorship contribution statement

**Kasturi Pusty:** Data curation, Formal analysis, Investigation, Methodology, Software, Validation, Visualization, Writing – original draft. **Kshirod Kumar Dash:** Conceptualization, Funding acquisition, Investigation, Methodology, Project administration, Resources, Software, Supervision, Validation, Visualization, Writing – original draft, Writing – review & editing. **Souvik Giri:** Data curation, Formal analysis, Investigation, Methodology, Software, Validation, Visualization, Writing – original draft. **G.V.S. Bhagya Raj:** Data curation, Formal analysis, Investigation, Methodology, Software, Validation, Visualization, Writing – original draft. **Ajita Tiwari:** Funding acquisition, Project administration, Validation, Writing – review & editing. **Ayaz Mukarram Shaikh:** Funding acquisition, Project administration, Resources, Validation, Writing – review & editing. **Kovács Béla:** Funding acquisition, Project administration, Validation, Writing – review & editing.

## Declaration of competing interest

The authors declare that they have no known competing financial interests or personal relationships that could have appeared to influence the work reported in this paper.
